# Mechanistic Insights into Arbuscular Mycorrhizal Fungi-Mediated Drought Stress Tolerance in Plants

**DOI:** 10.3390/ijms20174199

**Published:** 2019-08-27

**Authors:** Ali Bahadur, Asfa Batool, Fahad Nasir, Shengjin Jiang, Qin Mingsen, Qi Zhang, Jianbin Pan, Yongjun Liu, Huyuan Feng

**Affiliations:** 1 MOE Key Laboratory of Cell Activities and Stress Adaptation, School of Life Sciences, Lanzhou University, Lanzhou 730000, China; 2 State Key Laboratory of Grassland Agro-ecosystems, Institute of Arid Agroecology, School of Life Sciences, Lanzhou University, Lanzhou 730000, China; 3 MOE Key Laboratory of Western China’s Environmental Systems, College of Earth and Environmental Sciences, Lanzhou University, Lanzhou 730000, China; 4 Key Laboratory of Mollisols Agroecology, Northeast Institute of Geography and Agroecology, Chinese Academy of Sciences, Changchun 130102, China; 5 Key Laboratory of Vegetation Ecology, Ministry of Education, Institute of Grassland Science, Northeast Normal University, Changchun 130024, China

**Keywords:** arbuscular mycorrhizal fungi, drought tolerance mechanisms, phytohormones, biochemical responses, aquaporins

## Abstract

Arbuscular mycorrhizal fungi (AMF) establish symbiotic interaction with 80% of known land plants. It has a pronounced impact on plant growth, water absorption, mineral nutrition, and protection from abiotic stresses. Plants are very dynamic systems having great adaptability under continuously changing drying conditions. In this regard, the function of AMF as a biological tool for improving plant drought stress tolerance and phenotypic plasticity, in terms of establishing mutualistic associations, seems an innovative approach towards sustainable agriculture. However, a better understanding of these complex interconnected signaling pathways and AMF-mediated mechanisms that regulate the drought tolerance in plants will enhance its potential application as an innovative approach in environmentally friendly agriculture. This paper reviews the underlying mechanisms that are confidently linked with plant–AMF interaction in alleviating drought stress, constructing emphasis on phytohormones and signaling molecules and their interaction with biochemical, and physiological processes to maintain the homeostasis of nutrient and water cycling and plant growth performance. Likewise, the paper will analyze how the AMF symbiosis helps the plant to overcome the deleterious effects of stress is also evaluated. Finally, we review how interactions between various signaling mechanisms governed by AMF symbiosis modulate different physiological responses to improve drought tolerance. Understanding the AMF-mediated mechanisms that are important for regulating the establishment of the mycorrhizal association and the plant protective responses towards unfavorable conditions will open new approaches to exploit AMF as a bioprotective tool against drought.

## 1. Introduction

Drought stress, one of the major abiotic stresses, drastically affects crop production and jeopardizes food security globally [1]. Plants cope with drought deficit condition by recruiting drought avoidance and/or drought tolerance mechanisms, which include morphological, physiological, and molecular responses [2,3]. Water deficit condition negatively affects several aspects of plant physiology [4]. For instance, it uncouples photosynthesis, disorders the structure of enzymes, reduces nutrient uptake and/or transport to the shoot, therefore prompting a hormonal and nutritional imbalance in the plant [5,6,7]. In addition, drought stress results in osmotic stress that can lead to turgor loss, thereby, leading to inhibition in plant growth and development [5]. Drought stress also induces the production of reactive oxygen species (ROS), resulting in oxidative damage to carbohydrates, protein synthesis, lipid metabolisms, and alternatively leading to the membrane damage and cell death in plant tissues [8,9].

Plants greatly rely on root-associated microflora to mitigate various environmental stresses i.e., drought stress [10,11]. Amongst them, the arbuscular mycorrhizal fungi (AMF), which belong to the phylum Glomeromycota, establish a symbiotic relationship with the host plants. AMF take photosynthetic products, including carbohydrates, from the host plant roots [12,13]. AMF not only increase water and nutrient uptake to mitigate the negative impact of drought but also improve stomatal regulation [14]. For instance, improved stomatal conductance has been reported in AMF-inoculated *Poncirus trifoliata* and *Rosmarinus officinalis* plants under drought stress [15,16,17]. In addition, to an increase in water use efficiency (WUE), AMF regulate various mechanisms to reduce oxidative damage under drought stress and represent a promising avenue to improve next-generation agriculture [18]. In response to drought stress, the development of AMF-mediated mechanisms includes modifications in the content of plant hormones, such as strigolactones, jasmonic acid (JA), and abscisic acid (ABA), and improvement in plant water status by increasing hydraulic conductivity [19]. In *Solanum lycopersicum*, AMF enhanced the plant drought tolerance by regulating the 14-3-3 genes (*TFT1-TFT12*) in the ABA signaling pathway and improved the plant water relations [20].

The current review is a refreshing contrast on previous reports and unique in its approach as not only giving an up-to-date insight into present knowledge pertaining AMF-mediated drought stress tolerance at biochemical, molecular, and morphological levels but also examining the latest significant contributions by researchers on the role of AMF in improving different aspects of plant/crop performance under drought stress.

## 2. The Influence of Drought Stress on AMF Diversity and Growth

AMF attribute widely in the context of upgrading soil structure and its water holding capacity. AMF species diversity depends highly on the mode of new methodology and application [21]. In different ecosystems, approximately 250 AMF species have been recognized to date [22]. On one side, AMF favor enhanced tolerance-related responses to drought stress [23], which substantially improve plant growth and crop production [24,25]. On the contrary, there is a direct effect of water shortage on AMF development cycle, which hampers the AMF spore germination, colonization capacity, sporulation, and extra-radical hyphal elongation [1].

Over the modern decades, a major focus is to understand how AMF composition and diversity is affected by drought stress. The AMF species are found to be lower in the water-deprived soils, relative to normal soils, with a distribution of *Glomeraceae* as the “global family”. These AMF species show an opportunistic behavior because they utilize their energy mostly in the production of more descendants. In addition, AMF species have evolved characteristics that are advantageous in dry environments [26]. Some of the AMF isolates or individual species can tolerate drought stress and are extensively distributed [27]. Native AMF ecotypes have come up after a long span of adaptation to the soils of extreme characteristics [28]. A number of investigations pointed out that *Glomus* species are typical of semi-arid Mediterranean ecosystems and are able to grow under water deficit conditions [27,29,30,31,32,33].

## 3. AMF and Host Plant Association During Drought Stress

Plants respond to drought stress by adopting different strategies, which allow them to avoid stress and/or enhance drought tolerance. Although these modifications have vital significance to most of the plant species, they have not been confirmed as a common response in host plant species with different evolutionary trajectories [34,35,36]. These plant adaptive strategies allow the plant to withstand water-limiting conditions by maintaining a higher water status. Plant roots have highly plastic traits that can be modulated by AMF to enhance water uptake and/or minimize water loss. This dehydration tolerance is associated with survival and sustained physiological adaptations, when the leaf water potential is low, promote the ability of leaves to endure dehydration [37]. Fascinatingly, upon a perception of drought stress, most of the plants immediately ask for AMF help, by secreting rhizosphere signaling molecule (a class of phytohormones), so-called “strigolactone” [38]. Recently, the role of AMF inoculation to alleviate drought stress has been receiving great attention [39]. Mycorrhizal association with host plants increases the hydration status at whole-plant level, as characterized by leaf relative water content (LRWC) [40,41], while a detailed review of the literature shows that leaf water potential was not static in some experiments [42,43] (Table 1). Although the AMF colonization-mediated adaptive mechanisms in plants have been firmly established, several questions still remain open.

**Figure 1:**
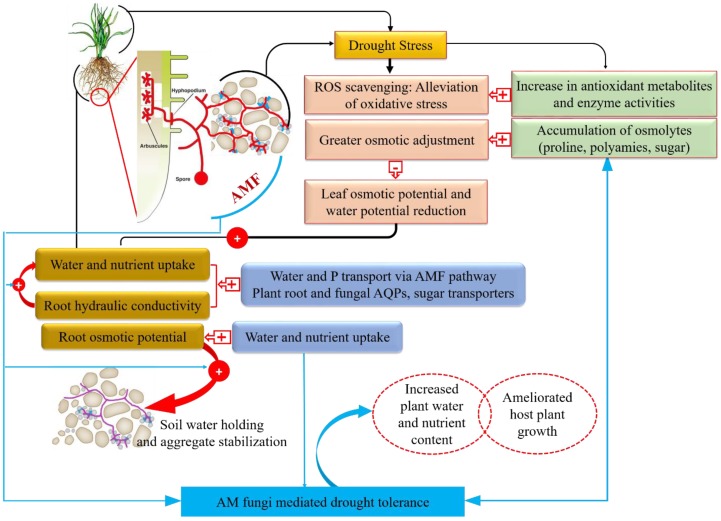
Arbuscular mycorrhizal fungi (AMF) symbiosis helps plants to maintain and regulate different processes in plants to cope with deleterious effects of drought stress, through either direct or indirect interaction, on plant growth performance. “+”and “−” symbols indicate an increase and a decrease in the production and accumulation of specific compounds.

**Figure 2:**
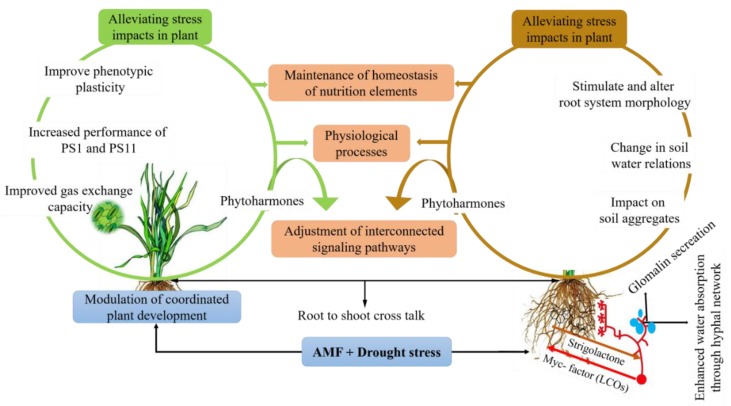
Schematic diagram showing a complex network of mechanisms mediated by AMF to alleviate drought stress symptoms in plants. Through the establishment of mycorrhizal symbiosis and/or exudation of specific compounds like strigolactones, plants adapt different strategies to alleviate deleterious effects of stress. This plant–AMF association alters root physiology and plant growth by acting on different physiological, biochemical and molecular mechanisms that essentially enhance water and nutrient uptake. Altered root physiology impacts root-to-shoot cross-talk and contributes to the maintenance of homeostasis water, hormones, and ions in the plant. This alleviates the drought-induced growth impairment, and in turn, plants adapt their phenotype according to stress conditions by regulating the expression of stress-related genes and proteins. AMF symbiosis helps maintain the plant in water uptake, producing a large amount of external mycelium and increasing the effective root surface area by fungal hyphae or by increasing the lateral root formation. The exchange of water and nutrients between the two partners takes place through the arbuscules formed within the root cells of the host plant.

Mycorrhizal association with the plants under drought stress enhances plant performance by improving plant growth [44], water status, and nutrient accumulation [45]. During this process, AMF-colonization improves the establishment of extensive hyphal networks and glomalin secretion, which in turn, assist in water and nutrient uptake, and thereby, enhance soil structure (Figure 1 and Figure 2) [46,47]. Besides, studies suggested that AMF develop drought-adaptive strategies through the extra-radical hyphae, and influence plant mechanisms, such as photosynthetic rate, root hydraulic conductivity, and root architecture [48,49,50]. AMF-mediated responses constitute an array of multifaceted mechanisms which include induction of drought-responsive genes and activation of different metabolic pathways. Studies to date have explored the vital role of AM symbiosis in ameliorating drought by up and down-regulating the numerous biochemical and physiological pathways. AMF amend the water regulation in the host plants by triggering hormonal signaling or by stimulating osmolytes. ABA is one of the non-nutritional mechanisms by which AM symbiosis regulates stomatal conductance and other physiological traits in response to drought stress [51].

In roots and shoots, the osmotic stress induced by drought is tolerated by host plant via altering biochemical responses which mostly comprise of enhanced metabolites biosynthesis, such as sugars and proline, that function as osmolytes [12,19]. These metabolic compounds contribute to reducing the osmotic potential, and thereby, the leaf water potential in drought exposed plants [59,60]. These lower levels of potentials allow mycorrhizal plants to sustain high organ hydration and turgor level which maintain overall physiological activities of the cells and especially linked to the photosynthetic apparatus [61]. AMF plants counteract water deficit-induced oxidative stress by enhancing the antioxidant compounds production which scavenges ROS and improves the antioxidant enzymatic activities. AMF root colonization increases root growth, hydraulic properties, and root architecture, and consequently, induces the development of a greatly functional root system to uptake water nutrients [23]. In the meanwhile, AMF hyphae in the soil establish the beneficial pathways for water and nutrient acquisition and transport, providing better beneficial exploitation of nutrient and water reservoirs in the soil, the area where only the AMF could grow, in turn surpassing nutrient and water depletion zones around the plant roots. AMF symbiosis activates the molecular mechanisms to cope with stress impacts, i.e., the activation of functional protein genes, such as aquaporins membrane transporters, and potentially, sugar and ion transporters in fungi and roots. Enhanced transport of root translates water and nutrient acquisition into improved hydration in the aboveground plant parts, thereby affects the biochemical and physiological processes. Moreover, AMF symbiosis improves the plant resistance to drought stress by secondary responses, such as enhancing the soil structural stability, in turn, enhancing the soil water retention (Figure 1).

## 4. Mechanisms of AMF-Mediated Drought Stress Tolerance

Drought stress is responsible for the deterioration in the soil and presents severe threats to agriculture worldwide. Most of the research over the last few years has focused on symbiotic mechanisms of AMF for protecting plants against drought stress, which verdict, symbiosis often marks an increased accumulation of osmoregulators, nutrient uptake, WUE and photosynthetic rate [57]. From a research viewpoint, efforts from both AMF and drought stress fields have improved our understanding of these mechanisms. In this regard, more recently, it was confirmed that AM symbiosis-specific downstream responses control a combination of morphological, biochemical, and physiological plant characteristics (Table 1). In this section of the current review, we will insight the most important mechanisms of AMF-mediated drought stress tolerance in plants.

### 4.1. AMF-Assisted Drought Stress Tolerance at Biochemical Level

The AMF function against water stress episodes and facilitate the drought-exposed plants by regulating their biochemical mechanisms, where two mechanisms are more pronounced to explain the low oxidative damage in AMF-inoculated plants. The first mechanism involves the direct water absorption by hyphae and its transfer to the host plant, increasing the water content and scavenging the generation of ROS, such as hydroxyl radicals (^·^OH), singlet oxygen (^1^O_2_), hydrogen peroxide (H_2_O_2_), and superoxide anion radical (O_2_^·−^) [55]. Oxidative stress is accompanied by drought stress in plants and develops due to the production of ROS (Figure 1) [62]. A great body of evidence exists, though, that shows the accumulation of ROS under drought stress causes damage in the structure of carbohydrates, lipids, proteins, and DNA, which ultimately leads to the membrane damage and cell death [9,63,64].

The second mechanism entails an enhancement in the production of enzymatic and non-enzymatic antioxidants induced by symbiotic association [52,65]. The capacity of the antioxidant machinery governed by enzymatic and non-enzymatic antioxidant [54,66] work in plants to control and scavenge ROS [54,65,67]. Enzymatic antioxidants include ascorbate peroxidases (APX), superoxide dismutase (SOD), catalase (CAT), glutathione reductase (GR), guaiacol peroxidase (G-POD) and glutathione peroxidase (GPX). The non-enzymatic antioxidants include glutathione (GSH), ascorbate (ASC), carotenoid, flavonoids, and tocopherol [7,68,69,70]. For example, AMF symbiosis lowered the oxidative stress in maize plants under drought conditions and was also observed to provide profit in terms of non-systematic oxidative stress [71] (Figure 1). However, further studies are required to explore the actual AMF mechanisms and functions involved in the production of antioxidants and alteration in ROS metabolism. The influence of AMF symbiosis on the antioxidant capacity has been established to correlate with an increased transcription levels of enzymatic antioxidants and/or ascorbate and glutathione biosynthesis components, signifying the complex transcriptional regulation of the antioxidant machinery [65,72]. Additionally, some studies have focused on proteomic approaches, whereby the proteomic replies specific to AMF in water-deficient plants will help elucidate how mycorrhization provokes nutrient uptake, plant growth, and stress-tolerance responses. Antioxidants act not only as a direct ROS scavenger but also as a key sensor of the cellular redox status, so they trigger a number of signaling events to tightly control cellular ROS levels.

Many types of signaling molecules have the potential to act under specific conditions as phytohormones including ethylene, ABA, cytokinins, salicylic acid (SA), jasmonic acid (JA), and auxin during the process of AMF symbiosis against drought stress (Table 1, Figure 2) [19,56,73,74]. Due to the considerations regarding the length of the current review, this section will only discuss recent progress to understand the signaling and communication events between AMF and the host plant that can be useful for a more precise characterization of the AMF plant symbiosis under drought stress. Readers are suggested to excellent reviews for understanding the role of AMF-dependent phytohormones against drought stress [19,24,39,75,76].

Development in symbiosis starts with the signaling that happens earlier to the physical contact between the symbionts, where both symbionts release biochemical signaling molecules which stimulate preparatory responses in the other [6,77]. Molecular dialogue begins by strigolactones, a group of carotenoid-based phytohormones, released by the plant, controlling various aspects of plant development [78]. Strigolactones are released into the rhizosphere, where these labile signaling molecules attract AMF to identify a specific host in their neighborhood during the pre-contact phase (Figure 2). AMF trigger oxidative metabolism, on recognition of strigolactones, which stimulates improvement of hyphal branching and growth, resulting in the occurrence of physical interaction with a host plant root and driving them to symbiosis [76,79,80] (Figure 2). Increased production of strigolactones has been reported in lettuce and tomato plants under drought in the presence of *Rhizophagus irregularis*, indicating that AMF symbiosis induces striglolactones biosynthesis [6]. The expression of two tomato genes, *SlCCD7* and *SlCCD8*, involved in the biosynthesis of striglolactones was quantified. The expression of *SlCCD8* was not altered, however, *SlCCD7* expression was clearly up-regulated by increasing severity of the drought stress in host roots. Known aspects about the AM symbiosis and strigolactones mode of action suggest that it alleviates the negative effects of drought by regulating the plant physiology and development. These findings confirm the potential use of arbuscular mycorrhizas as biofertilizers to sustain crop production and agriculture development under unfavorable conditions. Strigolactones involvement in protecting AMF host plants against drought stress leads to the questions, whether it is a common symbiosis signaling pathway in a diversity of plant species. However further study is required to reveal the intrinsic mechanisms of strigolactones modulated by AM symbiosis against drought stress.

AMF-induced enhanced drought tolerance has been communicated so far, which alleviates the negative effects of drought stress by altering the hormonal profiles. The underlying mechanisms involved in the production of signaling molecules interacting with the plant-fungus association remain greatly unidentified. The phytohormone, ABA, considered as the ‘abiotic stress hormone’, increases under drought in AMF symbiotic plants to cope with the respective stress (Table 1, Figure 2). The simultaneous increase in the expression of plant genes encoding D-myo-inositol-3-phosphate synthase (IPS) and 14-3-3-like protein GF14 (14-3GF), which were responsible for ABA signaling transduction, was found to be involved in the activation of 14-3-3 protein and aquaporins (GintAQPF1 and GintAQPF2) in *Rhizophagus intraradices*. These findings suggest that coexpression of IPS and 14-3GF is responsible for the synergistic actions of the symbiotic partners in enhancing plant drought tolerance [81]. Various studies highlighted the mechanistic insights about the enhanced production of ABA in AMF host plant to confer tolerance against drought stress [82,83,84].

Apart from drought tolerance responses, production of ABA is essential for an appropriate establishment of AM colonization in plant roots [85], with evidence of its impact on the formation and functioning of arbuscules [85,86]. Hence an enhanced ABA biosynthesis in the AM symbiosis stressed plants serves to improve drought tolerance, whereas meanwhile, it increases and establishes AM symbiosis. The continuous adjustment of plants’ ABA levels in response to AM symbiosis elucidates its critical importance in communicating drought tolerance (Figure 1 and Figure 2). During drought stress, AMF induce ABA biosynthesis which ultimately increase the ABA levels in plants and promotes the stomatal closer to minimize water loss by transpiration [18,86]. On the other hand, a reduction in ABA production or an alteration in ABA signaling pathways has been observed in the roots of *Glycyrrhiza uralensis* [7], when they were not exposed to drought stress [87,88]. As it is evident that ABA is the ‘abiotic stress hormone’ this reduced level of ABA may elucidate that mycorrhizal plants have enhanced tolerance against water stress as compared to non-mycorrhizal plants, which translates into improved plant performance and fitness. Knowledge regarding the role of ABA in regulating stomatal closure in AM-induced drought tolerance is scarce, therefore, further research is required to answer these questions.

Another impact of drought stress on plant tissues is recognized in the context, a loss in intracellular water and osmotic damage. Osmotic stresses are involved in the inhibition of cell growth by restricting cellular expansion and cell wall extension, leading to a decrease in plant growth and development (Figure 1). Production of free amino acids is the vital osmolytes function in the osmotic adjustment of plants [89]. AMF symbiosis improved the growth performance and osmotic adjustment in *Macadamia tetraphylla* L. by the accumulation of different compounds, such as soluble sugar, proline, and free amino acids, under drought [90]. Osmotic adjustment assists plants to sustain water potential gradient for water flow from soil into roots [90,91]. It has been reported that the root colonization by AMF in *Medicago sativa* L. induces proline accumulation in leaves and roots against water deficit condition [92]. In water-deficient plants, proline frequently functions as an osmoprotectant, as a solute for the security of enzymes and proteins from denaturation. It also functions as a sink for energy, as an alleviator of cell acidity, and as a hydroxyl radical scavenger to control redox potential [60,93]. The AM symbiosis affects the metabolic regulation of organic acids emitted by leaves under drought episodes and improves the energetic position of the plant and works as an assistant to mitigate the drought deficit.

Classically, soluble sugar accumulation for adjusting the osmotic potential in plants in response to drought stress establishes an important plant-protective mechanism (Figure 1) [94]. AMF symbiosis is able to alter the pattern of gene expression encoding for ∆ ^1^-pyrroline-5-carboxylate synthetase (p5cs) (showed lower p5cs transcript accumulation) in *Glycine max* and *Lactuca sativa* plants and protects host plants against drought [95]. Several studies have described the influence of AM symbiosis to maintain osmotic adjustments in terms of soluble sugar where AM-inoculation decreased the soluble sugars in drought-exposed plants, i.e., *Erythrina variegata* [35] and *Casuarina equisetifolia* [96] and improved the plant tolerance in host plants. On the contrary, many authors have documented the positive correlation between sugar accumulation and mycorrhization, which might be due to the sink effect on the fungus demanding sugars from plant shoot tissues [42,90]. The mechanisms involved in the mycorrhiza development, often lead to improved photosynthesis rate and accumulation in carbon compounds in the root systems of AMF colonized plants [1,97].

Proline is an amino acid, synthesized by plants in response to drought stress, and its activation results in an alteration in osmoprotectant, thereby helps in the maintenance of cell osmotic balance to mitigate the drought stress effects [98]. Additional evidence for proline suggests that it is actively involved in osmoregulation and scavenging of free radicals [90]. Proline acts as a molecular chaperone for stabilizing subcellular structures, thus protecting plant cells against damaging effects of drought episodes [99]. Multiple studies have demonstrated that in arbuscular mycorrhized plants proline accumulation leads to more enhanced stress tolerance than in non-AMF plants against drying conditions (Figure 1 and Figure 2) [23,51]. In contrast, in *Antirhinum majus* L. plants, although proline accumulation enhanced against drought stress, a lower level of proline was observed in AMF plants, as compared to non-AMF counterparts, and accounted to advance plant growth performance and biomass productivity [100]. In *Poncirus trifoliata* AMF decreased the activity of both P5CS and OAT in leaf, root, and total plant and increased tissue ProDH activity. This observation confirms that a decrease in proline accumulation in AMF plants might be derived from the integration of an inhibition of glutamate synthetic pathway of proline with an increase in proline degradation [101]. The AMF inoculation in water-deprived plants increased the accumulation of soluble nitrogenous compounds and free polyamines [102]. It can be concluded that fluctuations in proline levels by mycorrhization would be an adaptive strategy by plants in terms of drought avoidance or drought resistance to stress conditions. There is a profound gap to be abridged and coherent results concerning the expression patterns at molecular levels to be operated. To link and demarcate a lineage among genes specific for osmotic stress, proline accumulation, and delinking of AMF and non-AMF counterparts with proline synthesis need to be further analyzed.

During the onset and development of drought stress within a plant, all the major processes, including the plant metabolism, are affected. Beneficial effects of AMF symbiosis under drought stress may be due to alterations in basal energetic metabolism. Thus, changes in plant metabolism have also been reported in mycorrhizal plants grown at different drought stress levels. Maintenance of photosynthetic apparatus via the accumulation of protective molecules and osmolytes and/or the upregulation of antioxidant metabolism by AMF suggests the integration of biotechnological opportunities and their application for improving agricultural productivity.

### 4.2. AMF-Mediated Drought Stress Tolerance at Morphological Level

The AMF extend the capacity of plants for adaptation to the drought environment. The establishment of particular AMF member in the rhizosphere can be regarded as niche colonization. As mentioned, the impact of the AMF colonization is believed to rely heavily upon the survival and growth of the host plant [103], which also mediate tolerance via colonization which is beneficial for both the host plants and AMF. For example, three *Glomus species*, *G. macrocarpum*, *G. clarum*, and *G. etunicatum*, exhibited considerable tolerance to soil drying [104]. This could be a strategy for survival, implemented by AMF, because spores are a form of resistance propagules that can survive under adverse conditions (Table 1). To date, significant evidence has been accumulated on AMF survival in drought stress. AMF have been revealed to tolerate drought stress by implementing morphological adaptation (i.e., avoidance strategy). This adaptive root conductance is communicated with changes in the morphogenetic types of roots. AMF has the potential to reduce the meristem activity of root apices resulting in the formation of enhanced adventitious roots. These AMF-mediated modifications in root morphology may assist in maintaining nutrient uptake and water balance in the host plant under drought stress.

In response to drought stress, a stimulation of AMF development phenomenon has been revealed where it acts as an AMF defense reaction to alleviate the negative effects of water deficit. In a previous study, when AMF spores were treated by storage in different soil water potentials, *Glomus mosseae* and *G. deserticola* showed better infectivity, indicating that surrounding of spores might have a strong impact on its efficiency in root colonization [59]. Previously, [105] reported that spore germination of *Gigaspora margarita* was independent of drought episodes. Whenever drought stress has been investigated, AMF have demonstrated the ability to fix water uptake and improve plant nutrition through hyphal elongation. Moreover, better water status might trigger its action, resulting in increased activity and hydraulic conductivity of the roots [37,106,107]. Furthermore, AMF have the ability to perform the function of anastomosis (the capability to bring about vegetative cells’ inter-individual fusion) which is considered an imperative mechanism taking to AMF perseverance in dry conditions, particularly the anastomosis of disrupted mycelium to rebuild a linked network after facing water-deficit stress [108,109].

In contrast to above findings, even if AMF are ubiquitous in terrestrial ecosystems, including man-made habitats [110,111], many studies (Table 1) have revealed that the key stages in the AMF development cycle, such as spore germination, colonization, extraradical hyphal elongation, and sporulation, could be hindered by drought stress. A decrease in arbuscular and vesicle abundance in the roots of mycorrhiza was also observed in response to drought [112]. Several studies have described an inhibition in germinative hyphae elongation and spore germination upon drought stress [44,113]. The negative effect of drought on spore germination may influence the root colonization in the host plant. In fact, it is reported that drought stress can cause a serious decline in the formation of total root colonization (Table 1). This might be due to the inability of AMF spores to germinate and perceive an appropriate host plant root or might be due to the disruption at later steps in the process of colonization even after getting contact with the host [44,113,114]. Furthermore, this negative effect can be explicated either by an indirect impact because of inhibition in the root growth and or of a direct impact on the AMF development itself. Drought stress affects the post-symbiotic steps, including the post-colonization formation of extraradical mycelium and new spores.

### 4.3. Fungal Water Absorption and Transport Against Drought Stress in AMF Association

Uptake of water by the roots from the soil and its circulation across the whole plant parts are significant for all the physiological developments. Movement of water happens by a gradient-driven flow through membranes, a process which is regulated and mediated by water channels called aquaporins (AQPs) [7,58,115]. AQPs are a family of pore-forming integral membrane proteins that belong to the family of major intrinsic proteins (MIPs) and occur in all living cells/organisms and form large families in the plants. Based on the sequences of amino acid, AQPs are divided into five subfamilies: Tonoplast intrinsic proteins (TIPs), plasma membrane-intrinsic proteins (PIPs), and NOD26-like intrinsic proteins (NIPs) which were first recognized in legumes symbiosomes but they also exist in the endoplasmic reticulum and plasma membrane, small basic intrinsic proteins (SIPs) localized only in the endoplasmic reticulum (ER) of dicots, as well as uncharacterized intrinsic proteins (XIPs) found in the plasma membrane [18,116,117]. Plant AQPs play a key role in AM symbiosis and might respond differently to subjected drought stress and AMF colonization (Figure 1) [118,119]. Differential expression of genes coding for AQPs by AMF and drought stress is observed for some *PIPs* in plant roots [119,120]. For instance, the expression of two AQP genes *GintAQPF1* and *GintAQPF2* was significantly enhanced, in extraradical mycelia of *R. irregularis* and mycorrhizal roots in response to drought stress, thus supporting the existence of a direct AMF involvement in plant tolerance to water deprivation [121,122]. Consistently, the increased expression of AQPs genes in both root cortical cells containing arbuscules and extraradical mycelia under drought stress was reported, though possible mechanisms include direct water and nutrient uptake via extraradical hyphae, and better root system architecture [67,121,123,124]. This activity was inhibited by the presence of drought stress.

Previous studies clearly elucidated that AM symbiosis regulates the expression of key AQP genes and tightly programmed root plant water status as well as the hydraulic conductivity and tolerance under water deficiency [125,126]. In AM fungal-inoculated tomato plants, an enhancement in the water transport capacity of AMF roots, correlated with overexpression of NIP AQP-encoding gene (*LeNIP3;1*) [18]. Conversely, in another study a NIP AQP gene (*LjNIP1*) was up-regulated specifically in the arbuscule-containing cells in mycorrhizal roots of *Lotus japonicus* [127]. In contrast, under drought stress, *Funneliformis mosseae* exhibited higher expression levels of root *PtTIP1;2*, *PtTIP1;3*, and *PtTIP4;1* of *Poncirus trifoliata* L. and lower expression levels of root *PtTIP2;1* and *PtTIP5;1* [128]. It shows that root *TIPs* genes revealed diverse responses to mycorrhization, representing the multiple roles of AMF in water absorption under water stress.

Osmotic root hydraulic conductivity is defined as an observation of plant water flow through cell-to-cell connected pathways, where it is greatly allied to the water channels activity or the density of the plasma membrane in the cells [129]. Upon drought perception, plants usually show a decline in root hydraulic conductivity [125,130] perhaps as an adaptive mechanism to prevent water loss. These observations are similar to some other studies [58]. The absence of strong correlation between AQP genes expression and hydraulic conductivity suggests that, with the enhancement in hydraulic conductivity in plants inoculated by AMF, it might be owed to other processes like enlarged expression and/or action in plants AQP genes due to post-translational modifications of these proteins [131] or changes in the density or size of plasmodesmata in AMF roots. The increase in osmotic root hydraulic conductivity (*Lo*) in AM plants can be associated with an enhanced expression in fungal or plant AQPs [132]. It is noteworthy that under drought stress episodes, AMF plants enhanced maize growth, especially in the case of drought-sensitive cultivar. This beneficial effect of AMF symbiosis was linked to a better efficiency of PSII, higher membrane stability and lower lipid peroxidation [53,58,133]. It was investigated lately that differential regulation of PIP AQP genes in six rice varieties was linked to drought stress tolerance. Recently, [7] found that the expression of root AQP gene *PIP* was significantly upregulated by moderate water deficit in AMF roots. Additional, study on the in-vivo transport capacities by these AQP genes is necessary to understand the specific role of these proteins in the AMF-induced drought tolerance.

Some plant genes encoding AQPs were induced by AMF colonization, as shown for *RpPIP2;1* in *Robinia pseudoacacia* [134], which could be a way to upturn water flow in specific plant tissues, vital for host existence under drought stress. In this context, it was found that the AQP genes of AMF involve in water transport by mycorrhizal hyphae to the plant mate [122]. Likewise, in *Phaseolus vulgaris* and *Nicotiana tabacum* antisense type were colonized by AMF [135]. The AMF effects on AQP genes were reliant on the endogenous points of ABA in the host plant [10]. Though, in lettuce roots, the *GintAQP1* gene expression was decreased by water deficit, even if the root AMF was improved [119]. Advanced studies are required to analyze the AQP genes regulation in plants exposed to water episodes, which allocates both the transport of water from AMF hyphae to the roots and the roles of AQP genes in AMF-facilitated plant water transport. Differential regulation of AQP-encoding genes by AMF symbiosis and water stress episodes has been detected for some *PIPs* in *R. irregularis* [120,135]. Thus, AMF symbioses lead to a decreased or an increased expression in AQP genes, but the functionality of AQP in mycorrhizal systems is still poorly understood. The facilitated water transport in AMF symbiotic communication might also be connected to an enhanced membrane water permeability, demanding the up-regulation of AQPs under water deficit condition [136]. Some specific patterns of AQP regulation were identified in colonized roots, and these were linked to an overall enhancement in drought tolerance, as revealed by amended growth and water status of mycorrhizal plants [137].

## 5. Concluding Remarks and Future Perspectives

Drought episodes cause a significant reduction in agriculture productivity. Mycorrhizal symbiosis is a ubiquitous plant–microbe interaction, plays an important role in nutrient cycling that helps alleviate the deleterious effects of drought conditions by promoting plant performance and yield production. Consequently, this plant–fungus interaction has a great potential in an environmentally friendly sustainable agriculture. Surprisingly, although this symbiosis was established more than 450 million years, we are beginning to comprehend how its presence and functioning are maintained. In this review, we aimed at providing a brief remark towards the perspective of various protective mechanisms, including secondary metabolites, such as phytohormones and signaling molecules and metabolic pathways that play crucial roles to counteract drought under AMF. Among them, the classical approaches based on biochemical, physiological, and molecular responses have emerged as key mechanisms due to their multifunctional adaptive responses, open up a wide range of potentials in sustainable global food security. However, an in-depth understanding of the AMF-mediated drought tolerance mechanisms in plants is required to insight their full potential. Another important factor to consider is the signaling cross-talk that takes place during these adaptive practices. Deciphering how mycorrhizal plants act and interact in drought-protective processes will contribute to the design of responses to optimize AMF symbiosis not only in drought tolerance but also in other abiotic stresses.

## Figures and Tables

**Table 1:** Summary of drought stress effects on morphological, physiological, and biochemical differences in different experimental set-ups and plant species.

Plant Species	AMF Species	AMF Variables	Plant Variables			Reference
			Morphological	Physiological	Biochemical	
*Calotropis procera* Ait	*Glomus intraradices*	Col%^↑^		N^↑^, K^↑^	CAT^↑^, POD^↑^, APX^↑^, SOD^↑^	[52]
*Cynophala flexuosa* L.	AMF	Col% ^ns^		LRWC^↑^, PEUE^↑^ (Cycle 2), leaf construction cost^↑^ (Cycle 2), SLA^↑^ (Cycle 1)		[41]
*Glycine max* L.	AMF	Col%^↑^	Soil moisture (%)^↑^, LAI^↑^, growth performance^↑^	Pn^↑^, Leaf proline concentration^↓^		[44]
*Glycyrrhiza uralensis* Fisch. ex DC.	*Rhizophagus irregularis*	Col%^↑^, A%^↓^		Leaf proline concentrations^↑^, P^↑^, C:N^↑^, Pn^↑^, WUE^↑^, C:P^↓^, N:P^↓^	Root ABA^↓^	[7]
*Ipomoea batatas* (L.) Lam.	Commercial inoculum containing *Glomus sp.* and *Acaulospora sp.*		Plant growth^↑^, tubers per plant^↑^, tuber fresh weight^↑^	P content^↑^, soluble sugars^↑^, leaf osmotic potential^↑^, chlorophyll degradation^↓^, photosynthetic pigments^↑^, maximum quantum yield of PSII (Fv/Fm)^↑^, photon yield of PSII (ΦPSII) ^↑^, net photosynthetic rate^↑^	Proline^↑^	[53]
*Pelargonium graveolens* (L.) Herit.	*Funneliformis mosseae*, *Rhizophagus irregularis*	Col%^↓^		Essential oil content^↑^, oil yield^↑^	MDA^↓^, H_2_O_2_^↓^, CAT^↑^, APX^↑^, SOD^↑^, GPX^↑^	[54]
*Phoenix dactylifera* L.	*Funneliformis monosporum*, *Rhizophagus clarus*, *Glomus deserticola* ∆		Plant growth performance^↑^	Nutrient absorption^↑^, RWC^↑^, water potential^↑^, stomatal resistance^↓^		[25]
*Poncirus trifoliata* (L.)	*Diversispora versiformis*	Col%^↓^, hyphal length^↓^	Plant growth performance^↑^, root morphology^↑^	LWP^↓^	IAA^↑^, MeJA^↑^, NO^↑^	[37]
*Poncirus trifoliata* (L.)	*Funneliformis mosseae*	Col%^↓^	Shoot^↑^, root^↑^, total biomass^↑^, surface area of lateral roots^↑^		O_2_^·−↓^, H_2_O_2_^↓^, MDA^↓^	[55]
*Poncirus Trifoliata* (L.)	*Funneliformis mosseae*, *Paraglomus occultum*	Col%^↓^	Plant height^↑^, stem diameter^↑^, leaf number^↑^, leaf, stem, and root dry weight^↑^	LRWC^↑^	Leaf sucrose^↑^, glucose^↑^, fructose^↑^, leaf proline concentration^↓^	[40]
*Poncirus trifoliate* (L.)	*Funneliformis mosseae*	Col%^↓^	Plant height^↑^, shoot and root biomass^↑^, root hairs density^↑^, length^↑^, diameter^↑^		Root IAA^↑^	[56]
*Poncirus trifoliata* (L.)	*Funneliformis mosseae*, *Paraglomus occultum*	Col%^↓^	Root biomass^↑^, taproot length^↑^, number of lateral roots^↑^		Root sucrose^↓^, glucose^↑^, fructose^↑^ root sucrose relevant enzymes^↑^, root proline^↓^	[1]
*Solanum lycopersicum* L. mutant notabilis and its wild-type	*Rhizophagus intraradices*	Col%^↑^, Col%^↓^	Shoot biomass^↑^	Shoot and root P concentrations^↑^, WUE^↑^, Tr^↑^	ABA^↓^	[20]
*Solanum lycopersicum* L.	*Funneliformis mosseae*, *Rhizophagus intraradices*		Plant height^↑^, root fresh weight^↓^	Stomatal density^↑^, WUE^↑^, Tr^↑^	ABA^↓^, H_2_O_2_^↓^, proline^↑^	[18]
*Zea mays* L.	*Rhizophagus irregularis*	Col%^↓^	Plant growth^↑^	P^↑^, WUE^↑^, Rehydration rate^↑^, leaf moisture percentage^↑^	Proline^↑^, C:P^↓^, N:P^↓^, MDA^↓^	[57]
*Zea mays* L.	*Rhizophagus irregularis*	Col% ^ns^	Shoot dry weight^↑^, root dry weight^↑^	gs^↑^, Lpr^↑^, Lo^↑^	Root ABA^↑^	[58]

^↓^ and ^↓^ indicate increasing and decreasing responses. AMF, arbuscular mycorrhizal fungi; WUE, water use efficiency; Tr, transpiration rate; ABA, abscisic acid; Col, AMF colonization; P, phosphorus; C:N, carbon: nitrogen; Pn, photosynthetic rate; C:P, carbon: phosphorus ratio; N:P, nitrogen:phosphorus; LAI, leaf area index; LRWC, leaf relative water content; PEUE, photosynthetic energy use efficiency; SLA, specific leaf area; CAT, catalase; POD, peroxidase; APX, ascorbate peroxidase; SOD superoxide dismutase; N, nitrogen; K, potassium; gs, stomatal conductance; Lpr, hydrostatic root hydraulic conductivity; Lo, osmotic root hydraulic conductivity; IAA, indoleacetic acid; O_2_^·−^, superoxide radical; H_2_O_2_, hydrogen peroxide; MDA, malondialdehyde; LWP, leaf water potential; MeJA, methyl jasmonate; NO, nitric oxide; GPX, glutathione peroxidase; ns, non-significant.

## References

[1] Zhang F., Zou Y.N., Wu Q.S. (2018). Quantitative estimation of water uptake by mycorrhizal extraradical hyphae in citrus under drought stress. Sci. Hortic..

[2] Parent B., Hachez C., Redondo E., Simonneau T., Chaumont F., Tardieu F. (2009). Drought and abscisic acid effects on aquaporin content translate into changes in hydraulic conductivity and leaf growth rate: a trans-scale approach. Plant Physiol..

[3] Candar-Cakir B., Arican E., Zhang B. (2016). Small RNA and degradome deep sequencing reveals drought-and tissue-specific micrornas and their important roles in drought-sensitive and drought-tolerant tomato genotypes. Plant Biotechnol. J..

[4] Batool A., Akram N.A., Cheng Z.G., Lv G.C., Ashraf M., Afzal M., Xiong J.L., Wang J.Y., Xiong Y.C. (2019). Physiological and biochemical responses of two spring wheat genotypes to non-hydraulic root-to-shoot signalling of partial and full root-zone drought stress. Plant Physiol. Biochem..

[5] Selmar D., Kleinwächter M. (2013). Influencing the product quality by deliberately applying drought stress during the cultivation of medicinal plants. Ind. Crops Prod..

[6] Ruiz-Lozano J.M., Aroca R., Zamarreno A.M., Molina S., Andreo-Jimenez B., Porcel R., Garcia-Mina J.M., Ruyter-Spira C., Lopez-Raez J.A. (2016). Arbuscular mycorrhizal symbiosis induces strigolactone biosynthesis under drought and improves drought tolerance in lettuce and tomato. Plant Cell Environ..

[7] Xie W., Hao Z., Zhou X., Jiang X., Xu L., Wu S., Zhao A., Zhang X., Chen B. (2018). Arbuscular mycorrhiza facilitates the accumulation of glycyrrhizin and liquiritin in *Glycyrrhiza uralensis* under drought stress. Mycorrhiza.

[8] Atkinson N.J., Urwin P.E. (2012). The interaction of plant biotic and abiotic stresses: From genes to the field. J. Exp. Bot..

[9] Gill S.S., Tuteja N. (2010). Reactive oxygen species and antioxidant machinery in abiotic stress tolerance in crop plants. Plant Physiol. Biochem..

[10] Ruiz-Lozano J.M., Aroca R. (2010). Modulation of aquaporin genes by the arbuscular mycorrhizal symbiosis in relation to osmotic stress tolerance. Symbioses and Stress.

[11] Saia S., Amato G., Frenda A.S., Giambalvo D., Ruisi P. (2014). Influence of arbuscular mycorrhizae on biomass production and nitrogen fixation of berseem clover plants subjected to water stress. PLoS ONE.

[12] Latef A.A.H.A., Hashem A., Rasool S., Abd_Allah E.F., Alqarawi A.A., Egamberdieva D., Jan S., Anjum N.A., Ahmad P. (2016). Arbuscular mycorrhizal symbiosis and abiotic stress in plants: A review. J. Plant Biol..

[13] Smith S.E., Read D.J. (2008). Mycorrhizal Symbiosis.

[14] Auge R.M., Toler H.D., Saxton A.M. (2015). Arbuscular mycorrhizal symbiosis alters stomatal conductance of host plants more under drought than under amply watered conditions: A meta-analysis. Mycorrhiza.

[15] Wu Q.S., Xia R.X., Zou Y.N. (2006). Reactive oxygen metabolism in mycorrhizal and non-mycorrhizal citrus (*Poncirus trifoliata*) seedlings subjected to water stress. J. Plant Physiol..

[16] Ruiz-Sanchez M., Armada E., Munoz Y., Garcia de Salamone I.E., Aroca R., Ruiz-Lozano J.M., Azcon R. (2011). Azospirillum and arbuscular mycorrhizal colonization enhance rice growth and physiological traits under well-watered and drought conditions. J. Plant Physiol..

[17] Sanchez-Blanco M.J., Ferrandez T., Morales M.A., Morte A., Alarcon J.J. (2004). Variations in water status, gas exchange, and growth in *Rosmarinus officinalis* plants infected with *Glomus deserticola* under drought conditions. J. Plant Physiol..

[18] Chitarra W., Pagliarani C., Maserti B., Lumini E., Siciliano I., Cascone P., Schubert A., Gambino G., Balestrini R., Guerrieri E. (2016). Insights on the impact of arbuscular mycorrhizal symbiosis on tomato tolerance to water stress. Plant Physiol..

[19] Fernández-Lizarazo J.C., Moreno-Fonseca L.P. (2016). Mechanisms for tolerance to water-deficit stress in plants inoculated with arbuscular mycorrhizal fungi. A review. Agron. Colomb..

[20] Xu L., Li T., Wu Z., Feng H., Yu M., Zhang X., Chen B. (2018). Arbuscular mycorrhiza enhances drought tolerance of tomato plants by regulating the 14-3-3 genes in the ABA signaling pathway. Appl. Soil Ecol..

[21] Bahadur A., Jin Z., Jiang S., Chai Y., Zhang Q., Pan J., Liu Y., Feng H. (2019). Arbuscular mycorrhizal spores distribution across different ecosystems of Qinghai Tibetan Plateau. Pak. J. Bot..

[22] Opik M., Zobel M., Cantero J.J., Davison J., Facelli J.M., Hiiesalu I., Jairus T., Kalwij J.M., Koorem K., Leal M.E. (2013). Global sampling of plant roots expands the described molecular diversity of arbuscular mycorrhizal fungi. Mycorrhiza.

[23] Ouledali S., Ennajeh M., Zrig A., Gianinazzi S., Khemira H. (2018). Estimating the contribution of arbuscular mycorrhizal fungi to drought tolerance of potted olive trees (*Olea europaea*). Acta Physiol. Plant.

[24] Basu S., Rabara R.C., Negi S. (2018). AMF: The future prospect for sustainable agriculture. Physiol. Mol. Plant Pathol..

[25] Meddich A., Jaiti F., Bourzik W., Asli A.E., Hafidi M. (2015). Use of mycorrhizal fungi as a strategy for improving the drought tolerance in date palm (*Phoenix dactylifera*). Sci. Hortic..

[26] Sykorova Z., Ineichen K., Wiemken A., Redecker D. (2007). The cultivation bias: different communities of arbuscular mycorrhizal fungi detected in roots from the field, from bait plants transplanted to the field, and from a greenhouse trap experiment. Mycorrhiza.

[27] Stahl P.D., Christensen M. (1991). Population variation in the mycorrhizal fungus Glomus mosseae: Breadth of environmental tolerance. Mycol. Res..

[28] Sylvia D.M., Williams S.E., Bethlenfalvay G.T., Linderman R.D. (1992). Vesicualr arbuscular mycorrhizae and environmental stress. Mycorrhiza in Sustainable Agriculture.

[29] ÖPik M., Moora M., Liira J., Zobel M. (2006). Composition of root-colonizing arbuscular mycorrhizal fungal communities in different ecosystems around the globe. J. Ecol..

[30] Mohammad M.J., Hamad S.R., Malkawi H.I. (2003). Population of arbuscular mycorrhizal fungi in semi-arid environment of Jordan as influenced by biotic and abiotic factors. J. Arid Environ..

[31] Panwar J., Tarafdar J.C. (2006). Distribution of three endangered medicinal plant species and their colonization with arbuscular mycorrhizal fungi. J. Arid Environ..

[32] Tian H., Gai J.P., Zhang J.L., Christie P., Li X.L. (2009). Arbuscular mycorrhizal fungi associated with wild forage plants in typical steppe of eastern Inner Mongolia. Eur. J. Soil Biol..

[33] Verma N., Tarafdar J.C., Srivastava K.K., Panwar J. (2008). Arbuscular mycorrhizal (AM) diversity in *Prosopis cineraria* (L.) Druce under arid agroecosystems. Agric. Sci. China.

[34] Bray E.A. (1997). Plant responses to water deficit. Trends Plant. Sci..

[35] Manoharan P.T., Shanmugaiah V., Balasubramanian N., Gomathinayagam S., Sharma M.P., Muthuchelian K. (2010). Influence of AM fungi on the growth and physiological status of *Erythrina variegata* Linn. grown under different water stress conditions. Eur. J. Soil Biol..

[36] Fan Q.J., Liu J.H. (2011). Colonization with arbuscular mycorrhizal fungus affects growth, drought tolerance and expression of stress-responsive genes in *Poncirus trifoliata*. Acta Physiol. Plant.

[37] Zou Y.N., Wang P., Liu C.Y., Ni Q.D., Zhang D.J., Wu Q.S. (2017). Mycorrhizal trifoliate orange has greater root adaptation of morphology and phytohormones in response to drought stress. Sci. Rep..

[38] Oldroyd G.E.D. (2013). Speak, friend, and enter: Signalling systems that promote beneficial symbiotic associations in plants. Nat. Rev. Microbiol..

[39] Kumar A., Verma J.P. (2018). Does plant-microbe interaction confer stress tolerance in plants: A review?. Microbiol. Res..

[40] Wu H.H., Zou Y.N., Rahman M.M., Ni Q.D., Wu Q.S. (2017). Mycorrhizas alter sucrose and proline metabolism in trifoliate orange exposed to drought stress. Sci. Rep..

[41] Barros V., Frosi G., Santos M., Ramos D.G., Falcao H.M., Santos M.G. (2018). Arbuscular mycorrhizal fungi improve photosynthetic energy use efficiency and decrease foliar construction cost under recurrent water deficit in woody evergreen species. Plant Physiol. Biochem..

[42] Auge R.M. (2001). Water relations, drought and vesicular-arbuscular mycorrhizal symbiosis. Mycorrhiza.

[43] Wu Q.S., Zou Y.N., Wu Q.S. (2017). Arbuscular mycorrhizal fungi and tolerance of drought stress in plants. Arbuscular Mycorrhizas and Stress Tolerance of Plants.

[44] Pavithra D., Yapa N. (2018). Arbuscular mycorrhizal fungi inoculation enhances drought stress tolerance of plants. Groundw. Sustain. Develop..

[45] Kapoor R., Evelin H., Mathur P., Giri B., Tuteja N., Gill S.S. (2013). Arbuscular mycorrhiza: Approaches for abiotic stress tolerance in crop plants for sustainable agriculture. Plant Acclimation to Environmental Stress.

[46] Gong M., Tang M., Chen H., Zhang Q., Feng X. (2012). Effects of two Glomus species on the growth and physiological performance of *Sophora davidii* seedlings under water stress. New For..

[47] Pagano M.C., Miransari M. (2014). Drought stress and mycorrhizal plant. Use of Microbes for the Alleviation of Soil Stresses.

[48] Lee B.R., Muneer S., Avice J.C., Jung W.J., Kim T.H. (2012). Mycorrhizal colonisation and P-supplement effects on N uptake and N assimilation in perennial ryegrass under well-watered and drought-stressed conditions. Mycorrhiza.

[49] Gholamhoseini M., Ghalavand A., Dolatabadian A., Jamshidi E., Khodaei-Joghan A. (2013). Effects of arbuscular mycorrhizal inoculation on growth, yield, nutrient uptake and irrigation water productivity of sunflowers grown under drought stress. Agr. Water Manag..

[50] Ruiz-Lozano J.M., Gómez M., Azcón R. (1995). Influence of different Glomus species on the time course of physiological plant responses of lettuce to progressive drought stress periods. Plant Sci..

[51] Doubková P., Vlasáková E., Sudová R. (2013). Arbuscular mycorrhizal symbiosis alleviates drought stress imposed on *Knautia arvensis* plants in serpentine soil. Plant Soil.

[52] Bahmani M., Naghdi R., Kartoolinejad D. (2018). Milkweed seedlings tolerance against water stress: Comparison of inoculations with *Rhizophagus irregularis* and *Pseudomonas putida*. Environ. Technol..

[53] Yooyongwech S., Samphumphuang T., Tisarum R., Theerawitaya C., Chaum S. (2016). Arbuscular mycorrhizal fungi (AMF) improved water deficit tolerance in two different sweet potato genotypes involves osmotic adjustments via soluble sugar and free proline. Sci. Hortic..

[54] Amiri R., Nikbakht A., Etemadi N. (2015). Alleviation of drought stress on rose geranium [*Pelargonium graveolens* (L.) Herit.] in terms of antioxidant activity and secondary metabolites by mycorrhizal inoculation. Sci. Hortic..

[55] Huang Y.M., Zou Y.N., Wu Q.S. (2017). Alleviation of drought stress by mycorrhizas is related to increased root H_2_O_2_ efflux in trifoliate orange. Sci. Rep..

[56] Liu C.Y., Srivastava A.K., Wu Q.S. (2017). Mycorrhizal fungi regulate root responses and leaf physiological activities in Trifoliate orange. Not. Bot. Horti Agrobot. Cluj Napoca.

[57] Zhao R., Guo W., Bi N., Guo J., Wang L., Zhao J., Zhang J. (2015). Arbuscular mycorrhizal fungi affect the growth, nutrient uptake and water status of maize (*Zea mays* L.) grown in two types of coal mine spoils under drought stress. Appl. Soil Ecol..

[58] Quiroga G., Erice G., Aroca R., Chaumont F., Ruiz-Lozano J.M. (2017). Enhanced drought stress tolerance by the arbuscular mycorrhizal symbiosis in a drought-sensitive maize cultivar is related to a broader and differential regulation of host plant aquaporins than in a drought-tolerant cultivar. Front. Plant Sci..

[59] Wu Q.S., Srivastava A.K., Zou Y.N. (2013). AMF-induced tolerance to drought stress in citrus: A review. Sci. Hortic..

[60] Ruiz-Lozano J.M. (2003). Arbuscular mycorrhizal symbiosis and alleviation of osmotic stress. New perspectives for molecular studies. Mycorrhiza.

[61] Smith S.E., Facelli E., Pope S., Smith F.A. (2010). Plant performance in stressful environments: interpreting new and established knowledge of the roles of arbuscular mycorrhizas. Plant Soil.

[62] Demidchik V. (2015). Mechanisms of oxidative stress in plants: From classical chemistry to cell biology. Environ. Exp. Bot..

[63] Fobert P.R., Despres C. (2005). Redox control of systemic acquired resistance. Curr. Opin. Plant Biol..

[64] Rhoads D.M., Umbach A.L., Subbaiah C.C., Siedow J.N. (2006). Mitochondrial reactive oxygen species. Contribution to oxidative stress and interorganellar signaling. Plant Physiol..

[65] Abbaspour H., Saeidi-Sar S., Afshari H., Abdel-Wahhab M.A. (2012). Tolerance of Mycorrhiza infected pistachio (*Pistacia vera* L.) seedling to drought stress under glasshouse conditions. J. Plant Physiol..

[66] Mittler R. (2002). Oxidative stress, antioxidants and stress tolerance. Trends Plant Sci..

[67] Zou Y.N., Huang Y.M., Wu Q.S., He X.H. (2015). Mycorrhiza-induced lower oxidative burst is related with higher antioxidant enzyme activities, net H_2_O_2_ effluxes, and Ca^2+^ influxes in trifoliate orange roots under drought stress. Mycorrhiza.

[68] Latef A.A.H.A., Chaoxing H. (2011). Effect of arbuscular mycorrhizal fungi on growth, mineral nutrition, antioxidant enzymes activity and fruit yield of tomato grown under salinity stress. Sci. Hortic..

[69] Chang M., Liao L., Lin J., Liu Z., Hsu Y., Lee T. (2012). Modulation of antioxidant defense system and NADPH oxidase in *Pluchea indica* leaves by water deficit stress. Bot. Stud..

[70] Essahibi A., Benhiba L., Babram M.A., Ghoulam C., Qaddoury A. (2017). Influence of arbuscular mycorrhizal fungi on the functional mechanisms associated with drought tolerance in carob (*Ceratonia siliqua* L.). Trees.

[71] Barzana G., Aroca R., Ruiz-Lozano J.M. (2015). Localized and non-localized effects of arbuscular mycorrhizal symbiosis on accumulation of osmolytes and aquaporins and on antioxidant systems in maize plants subjected to total or partial root drying. Plant Cell Environ..

[72] Marulanda A., Porcel R., Barea J.M., Azcon R. (2007). Drought tolerance and antioxidant activities in lavender plants colonized by native drought-tolerant or drought-sensitive Glomus Species. Microb. Ecol..

[73] Miransari M., Abrishamchi A., Khoshbakht K., Niknam V. (2014). Plant hormones as signals in arbuscular mycorrhizal symbiosis. Crit. Rev. Biotechnol..

[74] Miransari M. (2010). Contribution of arbuscular mycorrhizal symbiosis to plant growth under different types of soil stress. Plant Biol..

[75] Lopez-Raez J.A. (2016). How drought and salinity affect arbuscular mycorrhizal symbiosis and strigolactone biosynthesis?. Planta.

[76] Pandey A., Sharma M., Pandey G.K. (2016). Emerging roles of strigolactones in plant responses to stress and development. Front. Plant Sci..

[77] Chabaud M., Genre A., Sieberer B.J., Faccio A., Fournier J., Novero M., Barker D.G., Bonfante P. (2011). Arbuscular mycorrhizal hyphopodia and germinated spore exudates trigger Ca^2+^ spiking in the legume and nonlegume root epidermis. New Phytol..

[78] Lopez-Obando M., Ligerot Y., Bonhomme S., Boyer F.D., Rameau C. (2015). Strigolactone biosynthesis and signaling in plant development. Development.

[79] Kretzschmar T., Kohlen W., Sasse J., Borghi L., Schlegel M., Bachelier J.B., Reinhardt D., Bours R., Bouwmeester H.J., Martinoia E. (2012). A petunia ABC protein controls strigolactone-dependent symbiotic signalling and branching. Nature.

[80] Mori N., Nishiuma K., Sugiyama T., Hayashi H., Akiyama K. (2016). Carlactone-type strigolactones and their synthetic analogues as inducers of hyphal branching in arbuscular mycorrhizal fungi. Phytochemistry.

[81] Li T., Sun Y., Ruan Y., Xu L., Hu Y., Hao Z., Zhang X., Li H., Wang Y., Yang L. (2016). Potential role of D-*myo*-inositol-3-phosphate synthase and 14-3-3 genes in the crosstalk between *Zea mays* and *Rhizophagus intraradices* under drought stress. Mycorrhiza.

[82] Calvo-Polanco M., Sánchez-Romera B., Aroca R., Aroca R. (2013). Arbuscular mycorrhizal fungi and the tolerance of plants to drought and salinity. Symbiotic Endophytes.

[83] Hong J.H., Seah S.W., Xu J. (2013). The root of ABA action in environmental stress response. Plant Cell Rep..

[84] Martin-Rodriguez J.A., Huertas R., Ho-Plagaro T., Ocampo J.A., Tureckova V., Tarkowska D., Ludwig-Muller J., Garcia-Garrido J.M. (2016). Gibberellin-abscisic acid balances during arbuscular mycorrhiza formation in tomato. Front. Plant Sci..

[85] Herrera-Medina M.J., Steinkellner S., Vierheilig H., Bote J.A.O., Garcia Garrido J.M. (2007). Abscisic acid determines arbuscule development and functionality in the tomato arbuscular mycorrhiza. New Phytol..

[86] Pozo M.J., Lopez-Raez J.A., Azcon-Aguilar C., Garcia-Garrido J.M. (2015). Phytohormones as integrators of environmental signals in the regulation of mycorrhizal symbioses. New Phytol..

[87] Esch H., Hundeshagen B., Sehneider-Poetsch H., Bothe H. (1994). Demonstration of abscisic acid in spores and hyphae of the arbuscular-mycorrhizal fungus Glomus and in the NE-fixing cyanobacterium *Anabaena variabilis*. Plant Sci..

[88] Danneberg G., Latus C., Zimmer W., Hundeshagen B., Schneider-Poetsch H., Bothe H. (1993). Influence of vesicular-arbuscular mycorrhiza on phytohormone balances in maize (*Zea mays* L.). J. Plant Physiol..

[89] Bheemareddy V.S., Lakshman H.C. (2011). Effect of AM fungus *Glomus fasciculatumon* metabolite accumulation in four varieties of *Triticum aestivum* L. under short-term water stress. Vegetos.

[90] Yooyongwech S., Phaukinsang N., Cha-um S., Supaibulwatana K. (2013). Arbuscular mycorrhiza improved growth performance in *Macadamia tetraphylla* L. grown under water deficit stress involves soluble sugar and proline accumulation. Plant Growth Regul..

[91] Zhang B., Chang S.X., Anyia A.O. (2015). Mycorrhizal inoculation and nitrogen fertilization affect the physiology and growth of spring wheat under two contrasting water regimes. Plant Soil.

[92] Goicoechea N., Szalai G., Antolín M.C., Sánchez-Díaz M., Paldi E. (1998). Influence of arbuscular mycorrhizae and Rhizobium on free polyamines and proline levels in water-stressed alfalfa. J. Plant Physiol..

[93] Chaves M., Maroco J., Pereira J. (2003). Understanding plant responses to drought- from genes to the whole plant. Funct. Plant Biol..

[94] Bakr J., Pék Z., Helyes L., Posta K. (2018). Mycorrhizal inoculation alleviates water deficit impact on field-grown processing tomato. Pol. J. Environ. Stud..

[95] Porcel R., Azcón R., Ruiz-Lozano J.M. (2004). Evaluation of the role of genes encoding for Δ1-pyrroline-5-carboxylate synthetase (P5CS) during drought stress in arbuscular mycorrhizal *Glycine max* and *Lactuca sativa* plants. Physiol. Mol. Plant Pathol..

[96] Zhang H.H., Tang M., Chen H., Zheng C.L., Niu Z.C. (2010). Effect of inoculation with AM fungi on lead uptake, translocation and stress alleviation of *Zea mays* L. seedlings planting in soil with increasing lead concentrations. Eur. J. Soil Biol..

[97] Finlay R., Söderström B., Allen M. (1992). Mycorrhiza and carbon flow to the soil. Mycorrhizal Functioning: An Integrative Plant-Fungal Process.

[98] Koyro H.W., Ahmad P., Geissler N. (2012). Abiotic Stress Responses in Plants: An Overview.

[99] De Carvalho K., de Campos M.K., Domingues D.S., Pereira L.F., Vieira L.G. (2013). The accumulation of endogenous proline induces changes in gene expression of several antioxidant enzymes in leaves of transgenic Swingle citrumelo. Mol. Biol. Rep..

[100] Asrar A.A., Abdel-Fattah G.M., Elhindi K.M. (2012). Improving growth, flower yield, and water relations of snapdragon (*Antirhinum majus* L.) plants grown under well-watered and water-stress conditions using arbuscular mycorrhizal fungi. Photosynthetica.

[101] Zou Y.N., Wu Q.S., Huang Y.M., Ni Q.D., He X.H. (2013). Mycorrhizal-mediated lower proline accumulation in *Poncirus trifoliata* under water deficit derives from the integration of inhibition of proline synthesis with increase of proline degradation. PLoS ONE.

[102] Rapparini F., Peñuelas J., Miransari M. (2014). Mycorrhizal fungi to alleviate drought stress on plant growth. Use of Microbes for the Alleviation of Soil Stresses.

[103] Nasim G., Ashraf M., Ozturk M., Ahmad M.S.A. (2010). The role of arbuscualr mycorrhizae in inducing resistance to drought and salinity stress in crops. Plant Adaptation and Phytoremediation.

[104] Sylvia D.M., Schenck N.C. (1983). Application of superphosphate to mycorrhizal plants stimulates sporulation of phosphorus-tolerant vesicular-arbuscular mycorrhizal fungi. New Phytol..

[105] Douds D.D., Schenck N.S. (1991). Germination and hyphal growth of Vam fungi during and after storage in soil at five matric potentials. Soil Biol. Biochem..

[106] Liu T., Sheng M., Wang C.Y., Chen H., Li Z., Tang M. (2015). Impact of arbuscular mycorrhizal fungi on the growth, water status, and photosynthesis of hybrid poplar under drought stress and recovery. Photosynthetica.

[107] Zhu X.C., Song F.B., Liu S.Q., Liu T.D., Zhou X. (2012). Arbuscular mycorrhizae improves photosynthesis and water status of *Zea mays* L. under drought stress. Plant Soil Environ..

[108] Avio L., Pellegrino E., Bonari E., Giovannetti M. (2006). Functional diversity of arbuscular mycorrhizal fungal isolates in relation to extraradical mycelial networks. New Phytol..

[109] de la Providencia I.E., de Souza F.A., Fernandez F., Delmas N.S., Declerck S. (2005). Arbuscular mycorrhizal fungi reveal distinct patterns of anastomosis formation and hyphal healing mechanisms between different phylogenic groups. New Phytol..

[110] Enkhtuya B., Rydlová J., Vosátka M. (2002). Effectiveness of indigenous and nonindigenous isolates of arbuscular mycorrhizal fungi in soils from degraded ecosystems and man-made habitats. Appl. Soil Ecol..

[111] Lenoir I., Fontaine J., Lounes-Hadj Sahraoui A. (2016). Arbuscular mycorrhizal fungal responses to abiotic stresses: A review. Phytochemistry.

[112] Huang Z., Zou Z., He C., He Z., Zhang Z., Li J. (2010). Physiological and photosynthetic responses of melon (*Cucumis melo* L.) seedlings to three Glomus species under water deficit. Plant Soil.

[113] Estaun M.V. (1989). Effect of sodium chloride and mannitol on germination and hyphal growth of the vesicular-arbuscular mycorrhizal fungus Glomus mosseae. Agric. Ecosyst. Environ..

[114] Debiane D., Garçon G., Verdin A., Fontaine J., Durand R., Shirali P., Grandmougin-Ferjani A., Lounès-Hadj Sahraoui A. (2009). Mycorrhization alleviates benzo[a]pyrene-induced oxidative stress in an in vitro chicory root model. Phytochemistry.

[115] Nehls U., Dietz S. (2014). Fungal aquaporins: Cellular functions and ecophysiological perspectives. Appl. Microbiol. Biotechnol..

[116] Maurel C., Verdoucq L., Luu D.T., Santoni V. (2008). Plant aquaporins: Membrane channels with multiple integrated functions. Annu. Rev. Plant Biol..

[117] Reuscher S., Akiyama M., Mori C., Aoki K., Shibata D., Shiratake K. (2013). Genome-wide identification and expression analysis of aquaporins in tomato. PLoS ONE.

[118] Marjanovic Z., Uehlein N., Kaldenhoff R., Zwiazek J.J., Weiss M., Hampp R., Nehls U. (2005). Aquaporins in poplar: What a difference a symbiont makes!. Planta.

[119] Aroca R., Porcel R., Ruiz-Lozano J.M. (2007). How does arbuscular mycorrhizal symbiosis regulate root hydraulic properties and plasma membrane aquaporins in *Phaseolus vulgaris* under drought, cold or salinity stresses?. New Phytol..

[120] Porcel R., Aroca R., Azcon R., Ruiz-Lozano J.M. (2006). PIP aquaporin gene expression in arbuscular mycorrhizal *Glycine max* and *Lactuca sativa* plants in relation to drought stress tolerance. Plant Mol. Biol..

[121] Li T., Hu Y.J., Hao Z.P., Li H., Wang Y.S., Chen B.D. (2013). First cloning and characterization of two functional aquaporin genes from an arbuscular mycorrhizal fungus *Glomus intraradices*. New Phytol..

[122] Li Y., Zou Y.N., Wu Q.S. (2013). Effects of *Diversispora spurca* inoculation on growth, root system architecture and chlorophyll contents of four citrus genotype. Int. J. Agric. Biol..

[123] Neumann E., Schmid B., Romheld V., George E. (2009). Extraradical development and contribution to plant performance of an arbuscular mycorrhizal symbiosis exposed to complete or partial rootzone drying. Mycorrhiza.

[124] Zou Y.N., Srivastava A.K., Ni Q.D., Wu Q.S. (2015). Disruption of mycorrhizal extraradical mycelium and changes in leaf water status and soil aggregate stability in rootbox-grown trifoliate orange. Front. Microbiol..

[125] Aroca R., Porcel R., Ruiz-Lozano J.M. (2012). Regulation of root water uptake under abiotic stress conditions. J. Exp. Bot..

[126] Barzana G., Aroca R., Bienert G.P., Chaumont F., Ruiz-Lozano J.M. (2014). New insights into the regulation of aquaporins by the arbuscular mycorrhizal symbiosis in maize plants under drought stress and possible implications for plant performance. Mol. Plant Microbe Interact..

[127] Giovannetti M., Balestrini R., Volpe V., Guether M., Straub D., Costa A., Ludewig U., Bonfante P. (2012). Two putative-aquaporin genes are differentially expressed during arbuscular mycorrhizal symbiosis in *Lotus japonicus*. BMC Plant Biol..

[128] Jia-Dong H., Tao D., Hui-Hui W., Ying-Ning Z., Qiang-Sheng W., Kamil K. (2019). Mycorrhizas induce diverse responses of root TIP aquaporin gene expression to drought stress in trifoliate orange. Sci. Hortic..

[129] Tyerman S.D., Bohnert H.J., Maurel C., Steudle E., Smith J.A.C. (1999). Plant aquaporins: Their molecular biology, biophysics and significance for plant water relations. J. Exp. Bot..

[130] Javot H., Maurel C. (2002). The role of aquaporins in root water uptake. Ann. Bot..

[131] Chaumont F., Tyerman S.D. (2014). Aquaporins: Highly regulated channels controlling plant water relations. Plant Physiol..

[132] Sanchez-Romera B., Ruiz-Lozano J.M., Zamarreno A.M., Garcia-Mina J.M., Aroca R. (2016). Arbuscular mycorrhizal symbiosis and methyl jasmonate avoid the inhibition of root hydraulic conductivity caused by drought. Mycorrhiza.

[133] Grondin A., Mauleon R., Vadez V., Henry A. (2016). Root aquaporins contribute to whole plant water fluxes under drought stress in rice (*Oryza sativa* L.). Plant Cell Environ..

[134] He D., Xiang X., He J.S., Wang C., Cao G., Adams J., Chu H. (2016). Composition of the soil fungal community is more sensitive to phosphorus than nitrogen addition in the alpine meadow on the Qinghai-Tibetan Plateau. Biol. Fertil. Soils.

[135] Aroca R., Bago A., Sutka M., Paz J.A., Cano C., Amodeo G., Ruiz-Lozano J.M. (2009). Expression analysis of the first arbuscular mycorrhizal fungi aquaporin described reveals concerted gene expression between salt-stressed and nonstressed mycelium. Mol. Plant Microbe Interact..

[136] Yu Q., Hu Y., Li J., Wu Q., Lin Z. (2005). Sense and antisense expression of plasma membrane aquaporin BnPIP1 from *Brassica napus* in tobacco and its effects on plant drought resistance. Plant Sci..

[137] Ruiz-Lozano J.M., Porcel R., Aroca R. (2012). Regulation by arbuscular mycorrhizae of the integrated physiological response to salinity in plants: New challenges in physiological and molecular studies. J. Exp. Bot..

